# Supplementary material for: AffyMAPSDetector: a software tool to characterize Affymetrix GeneChip™ expression arrays with respect to SNPs
Source: BMC Bioinformatics. 2007 Jul 30;8:276. doi: 10.1186/1471-2105-8-276 (PMC1959249; doi:10.1186/1471-2105-8-276)
Supplement: Additional file 13 — AffyMAPSDetector v1 source code. AffyMAPSDetector source code. [file 1471-2105-8-276-S13.zip › AffyMAPSDetector_v1_src/doc/affychip/snpdetector/model/GeneInfo.html]

GeneInfo


|  |  |  |  |
| --- | --- | --- | --- |
| **Overview** \| **Package** \| **Class** \| **Use** \| **Tree** \| **Deprecated** \| **Index** \| **Help** | | |  |
| **PREV CLASS**   **NEXT CLASS** | **FRAMES**    **NO FRAMES**     **All Classes** |  |  |
| SUMMARY: NESTED \| FIELD \| CONSTR \| METHOD | DETAIL: FIELD \| CONSTR \| METHOD |  |  |


---


## affychip.snpdetector.model Class GeneInfo

```
java.lang.Object
  affychip.snpdetector.model.GeneInfo
```

---

public class **GeneInfo** extends java.lang.Object

Title: Probe SNP Detector

Description: This class captures the gene information such as "Nucleotide
Accession Number", "GI", "Locus Link ID", and "mRNA Sequence".

Copyright: Copyright (c) 2004

Company: George Mason University

**Version:**
:   1.1

**Author:**
:   Sunita Kumari

---

|  |  |
| --- | --- |
| **Field Summary** | |
| `static java.lang.String` | `delimiter` |
| `static java.lang.String[]` | `HEADER` |
| `static org.apache.log4j.Logger` | `logger` |


**Constructor Summary**

`GeneInfo(java.lang.String probesetId,
java.lang.String accNo,
java.lang.String locusId)`


|  |  |
| --- | --- |
| **Method Summary** | |
| `void` | `addSnpInfo(SnpInfo snpInfo)` |
| `java.lang.String` | `getDescription()` |
| `java.lang.String` | `getGenBankAccNo()` |
| `static java.lang.String` | `getHeader()` |
| `java.lang.String` | `getLocusId()` |
| `java.lang.String` | `getProbesetId()` |
| `java.lang.String` | `getRefSeqAccNo()` |
| `java.lang.String` | `getSequence()` |
| `SnpInfo` | `getSnpInfoFor(java.lang.String rsNo)`             This method return the `SnpInfo` object for a given rs-cluster number. |
| `SnpInfo[]` | `getSnpInfos()` |
| `java.lang.String[]` | `getSnps()` |
| `boolean` | `isRefSeq()` |
| `void` | `reset()`             This method exists mainly to circumvent memory issues. |
| `void` | `setDescription(java.lang.String desc)` |
| `void` | `setProbeLocationIndicesFor(Probe aProbe)`             This method finds starting index, ending index and index of 13th position of a given probe sequence relative to this gene's mRNA sequence. |
| `void` | `setRefSeqAccNo(java.lang.String refSeqAccNo)` |
| `void` | `setSequence(java.lang.String seq)` |
| `java.lang.String` | `toString()` |

**Methods inherited from class java.lang.Object**

`clone, equals, finalize, getClass, hashCode, notify, notifyAll, wait, wait, wait`

**Field Detail**

### HEADER

```
public static final java.lang.String[] HEADER
```

---


### delimiter

```
public static java.lang.String delimiter
```

---


### logger

```
public static org.apache.log4j.Logger logger
```


**Constructor Detail**

### GeneInfo

```
public GeneInfo(java.lang.String probesetId,
                java.lang.String accNo,
                java.lang.String locusId)
```


**Method Detail**

### isRefSeq

```
public boolean isRefSeq()
```

---


### addSnpInfo

```
public void addSnpInfo(SnpInfo snpInfo)
```

---


### getSnpInfos

```
public SnpInfo[] getSnpInfos()
```

---


### getSnpInfoFor

```
public SnpInfo getSnpInfoFor(java.lang.String rsNo)
```

:   This method return the `SnpInfo` object for a given rs-cluster
    number. If there is no SnpInfo with the given rs# then null is returned.

    :   **Parameters:**: `rsNo` - - reference snp (RS) cluster id e.g. rs7266 or 7266 (both are valid input to this method). **Returns:**: requested SnpInfo if found, null otherwise.

---


### setProbeLocationIndicesFor

```
public void setProbeLocationIndicesFor(Probe aProbe)
```

:   This method finds starting index, ending index and index of 13th position
    of a given probe sequence relative to this gene's mRNA sequence. If the
    probe's mismatch position's index (i.e. interrogation position) does not
    coinside with the one computed by this method, then the probe's
    interrogation postion is corrected.

    :   **Parameters:**: `aProbe` - **Returns:**

---


### setRefSeqAccNo

```
public void setRefSeqAccNo(java.lang.String refSeqAccNo)
```

:   **Parameters:**: `refSeqAccNo` - The refSeqAccNo to set.

---


### getRefSeqAccNo

```
public java.lang.String getRefSeqAccNo()
```

:   **Returns:**: Returns the refSeqAccNo.

---


### getDescription

```
public java.lang.String getDescription()
```

:   **Returns:**: Returns the refSeqGi.

---


### setDescription

```
public void setDescription(java.lang.String desc)
```

---


### getGenBankAccNo

```
public java.lang.String getGenBankAccNo()
```

---


### getLocusId

```
public java.lang.String getLocusId()
```

---


### getProbesetId

```
public java.lang.String getProbesetId()
```

---


### setSequence

```
public void setSequence(java.lang.String seq)
```

---


### getSequence

```
public java.lang.String getSequence()
```

---


### getSnps

```
public java.lang.String[] getSnps()
```

---


### toString

```
public java.lang.String toString()
```

---


### getHeader

```
public static java.lang.String getHeader()
```

---


### reset

```
public void reset()
```

:   This method exists mainly to circumvent memory issues. AffyMapsDetector
    can possibly run out of memory as it accumulates SNP information and
    Gene information (e.g. mRNA seq.). Once this information is used and
    written out to an appropriate output file, it is OK to free the memory
    occupied by it. *CAUTION !!!* Use this method with care. It should
    be called *ONLY* after all the information captured by it is
    properly used and it is not required any more.


---


|  |  |  |  |
| --- | --- | --- | --- |
| **Overview** \| **Package** \| **Class** \| **Use** \| **Tree** \| **Deprecated** \| **Index** \| **Help** | | |  |
| **PREV CLASS**   **NEXT CLASS** | **FRAMES**    **NO FRAMES**     **All Classes** |  |  |
| SUMMARY: NESTED \| FIELD \| CONSTR \| METHOD | DETAIL: FIELD \| CONSTR \| METHOD |  |  |


---
